# Lung Transplantation in Patients with High Lung Allocation Scores in the US: Evidence for the Need to Evaluate Score Specific Outcomes

**DOI:** 10.1155/2015/836751

**Published:** 2015-12-21

**Authors:** Jeremiah A. Hayanga, Alena Lira, Tedi Vlahu, Jingyan Yang, Jonathan K. Aboagye, Heather K. Hayanga, James D. Luketich, Jonathan D'Cunha

**Affiliations:** ^1^University of Pittsburgh Medical Center, Pittsburgh, PA 15213, USA; ^2^MedStar Washington Hospital Center, Washington, DC 20010, USA; ^3^DeVos Heart & Lung Transplantation Program, Spectrum Health-Michigan State University, Grand Rapids, MI 49504, USA; ^4^Columbia University, New York, NY 10032, USA; ^5^Perelman School of Medicine, University of Pennsylvania, Philadelphia, PA 19104, USA; ^6^Johns Hopkins Medical Institutions, Baltimore, MD 21205, USA

## Abstract

*Objective.* The lung allocation score (LAS) resulted in a lung transplantation (LT) selection process guided by clinical acuity. We sought to evaluate the relationship between LAS and outcomes.* Methods.* We analyzed Scientific Registry of Transplant Recipient (SRTR) data pertaining to recipients between 2005 and 2012. We stratified them into quartiles based on LAS and compared survival and predictors of mortality.* Results.* We identified 10,304 consecutive patients, comprising 2,576 in each LAS quartile (quartile 1 (26.3–35.5), quartile 2 (35.6–39.3), quartile 3 (39.4–48.6), and quartile 4 (48.7–95.7)). Survival after 30 days (96.9% versus 96.8% versus 96.0% versus 94.8%), 90 days (94.6% versus 93.7% versus 93.3% versus 90.9%), 1 year (87.2% versus 85.0% versus 84.8% versus 80.9%), and 5 years (55.4% versus 54.5% versus 52.5% versus 48.8%) was higher in the lower groups. There was a significantly higher 5-year mortality in the highest LAS group (HR 1.13, *p* = 0.030, HR 1.17, *p* = 0.01, and HR 1.17, *p* = 0.02) comparing quartiles 2, 3, and 4, respectively, to quartile 1.* Conclusion.* Overall, outcomes in recipients with higher LAS are worse than those in patients with lower LAS. These data should inform more individualized evidence-based discussion during pretransplant counseling.

## 1. Introduction

The lung allocation score (LAS) system was introduced in 2005 with the intention to increase patient survival rates and overall efficiency in transplant outcome and allocation. Specifically, the LAS system sought to decrease mortality due to end stage lung disease by prioritizing medical urgency. The system is hinged on an estimate of one-year survival for potential recipients and was designed to decrease waitlist mortality and further to maximize benefit to recipients and ensure most judicious, efficient, and equitable lung allocation [1–4]. The score, which ranges from 0 to 100, is calculated using a number of variables including respiratory function, health status, hemodynamic data, and diagnosis [2]. In principle, the higher the score, the higher the clinical acuity, primarily reflective of severity of lung disease.

Following the incorporation of the LAS system, recipients with higher scores became prioritized over those with lower scores. Critically ill potential recipients, who may have been overlooked prior to the LAS system, now receive donor lungs more expeditiously, theoretically allowing more effective mitigation of morbidity and mortality on the waiting list. However, despite the increased emphasis on the acutely ill candidates, several single and multicenter cohort studies have reported decreased short- and long-term survival in the higher LAS group (≥50) compared to the lower LAS (<50) group [5–10].

In this report, we use a national data registry to evaluate survival of recipients categorized into quartiles based on their LAS in order to report the most accurate survival estimates and to identify key factors that may contribute to mortality in recipients in our cohort beyond one year.

## 2. Methods

### 2.1. Study Population

We retrospectively examined the Scientific Registry of Transplant Recipients (SRTR) data files from the United Network for Organ Sharing (UNOS) database to identify recipients who had undergone LT between May 2005 and December 2012. This database maintains data elements reflecting donor characteristics, pretransplant recipient characteristics, and follow-up characteristics of posttransplant recipients. We included all consecutive adult LT recipients ≥18 years of age who underwent LT within the study period. We excluded those who had undergone multiple organ transplantation, those who received retransplantation, and those with missing information on pertinent variables. Both patient-level data and transplantation center data were provided in a deidentified format. Our institutional review board approved this study (#PRO1301170212).

The cohort was divided into quartiles based on their LAS scores: quartile 1 (26.3–35.5), quartile 2 (35.6–39.3), quartile 3 (39.4–48.6), and quartile 4 (48.7–95.7). The groups were compared using preselected recipient, donor, and transplant related characteristics, which included baseline demographic data and clinical descriptors such as primary pulmonary diagnosis, BMI, renal function, common comorbidities, respiratory support, and type of LT. Our primary outcome of interest was difference in survival between quartiles.

### 2.2. Statistical Analysis

Descriptive statistics were evaluated and expressed as mean ± SD for normally distributed continuous variables, median (25th to 75th percentiles) for nonnormally distributed continuous variables, and percentage for categorical variables at baseline. Difference among groups was tested by one-way ANOVA with Bonferroni correction for continuous variables and chi-square test for categorical variables. A Kaplan-Meier analysis was performed to estimate the 30-day, 90-day, 1-year, and 5-year survival after LT, respectively, and the differences in survival rate were compared using a log-rank test. A multivariate Cox proportional hazard model was fitted using recipient, donor, and transplant related variables to establish predictors of mortality. All the analyses were performed in Stata 13.0. Statistical tests were two sided and *p* values <0.05 were considered statistically significant.

## 3. Results

We identified 10,304 consecutive recipients, 2,576 in each quartile.  Table 1 describes the baseline demographic characteristics and common comorbidities such as diabetes mellitus or renal failure of the cohort. Patients within the higher LAS groups were more likely to bear a diagnosis of idiopathic pulmonary fibrosis, whereas COPD patients were likely to have lower LAS. Recipients in the higher LAS groups had overall more acute clinical status, as documented by the higher rates of mechanical ventilation or ECMO support prior to LT and the higher use of steroids at baseline. The primary pulmonary diagnosis in the lower LAS groups was predominantly COPD, whereas patients in the higher LAS group did not have clearly defined diagnosis. Higher LAS recipients were more likely to undergo double LT than the recipients in the lower LAS group.

**Figure 1:**
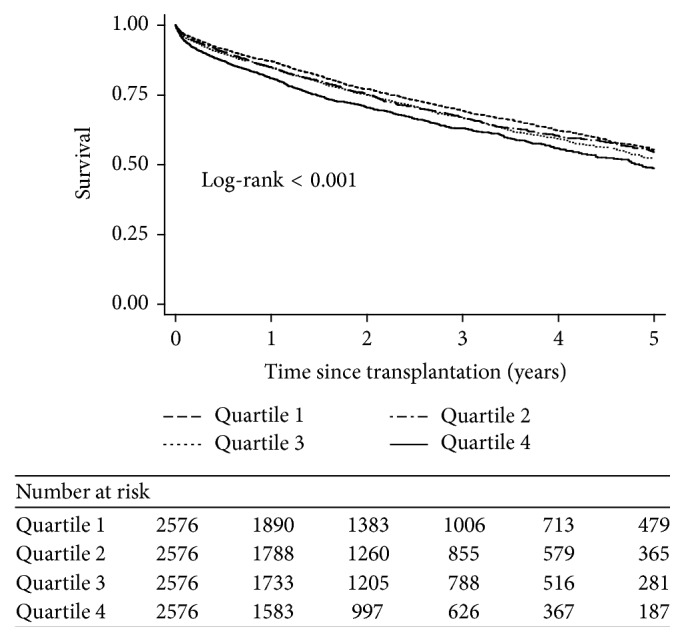
Kaplan-Meier survival curve after lung transplant, stratified by quartiles of lung allocation score.

Survival after 30 days, 90 days, 1 year, and 5 years was 96.9%, 94.6%, 87.2%, and 55.4.% respectively, in quartile 1, 96.8%, 93.7%, 85.0%, and 54.5%, respectively, in quartile 2, 96.0%, 93.3%, 84.8%, and 52.5%, respectively, in quartile 3, and 94.8%, 90.9%, 80.9%, and 48.8%, respectively, in quartile 4. A difference in survival between the groups was detected by a Kaplan-Meier analysis (Table 2, Figure 1). Furthermore, there was a significantly higher 5-year mortality among recipients with high LAS compared to lower LAS (HR 1.13, *p* = 0.030, HR 1.17, *p* = 0.01, and HR 1.17, *p* = 0.02) comparing quartiles 2, 3, and 4, respectively, to quartile 1 (Table 3). Other predictors of 5-year mortality included male gender (HR 1.10, 95% CI 1.02–1.18, *p* = 0.01), extremes of weight, both underweight (HR 1.02, 95% CI 0.94–1.11, *p* < 0.001) and obese (HR 1.16, 95% CI 1.04–1.30, *p* = 0.001), a diagnosis of pulmonary hypertension (HR 1.40, 95% CI 1.14–1.73, *p* = 0.002), ICU hospitalization (HR 1.96, 95% CI 1.69–2.27, *p* < 0.001), and ABO incompatibility (HR 1.17, 95% CI 1.03–1.32, *p* = 0.01). The use of mechanical ventilation (HR 1.08, 95% CI 0.92–1.27, *p* = 0.34) and ECMO (HR 1.34, 95% CI 0.96–1.84, *p* = 0.06) was found to be associated with an increased risk of 5-year mortality but these did not achieve statistical significance. An estimated glomerular filtration rate (GFR) of 60–90 (HR 0.77, 95% CI 0.68–0.88, *p* < 0.001) and >90 (HR 0.72, 95% CI 0.64–0.82, *p* < 0.001) was found to be protective compared to impaired renal function (GFR < 60). Similarly, double LT was found to be protective (HR 0.79, 95% CI 0.72–0.86, *p* < 0.001).

## 4. Discussion

The influence of the LAS system on outcomes has generated interest in assessing its ability to yield the best outcomes. Our current analysis confirms that, on a national scale, increasing LAS is significantly associated with increasing mortality. Multiple studies based on single center experiences have evaluated this but have reached varying conclusions [5, 8–14]. Many have reported decreased posttransplant survival corresponding with rising LAS [1, 8, 9, 11] and even identified inflection points at LAS of 46 and 60, beyond which risk of mortality is dramatically increased [7, 8]. This has led to increased criticism that LAS is conceptually more focused on reduction in waitlist mortality rather than long-term survival, challenging the appropriateness of preferential allocation of scarce organs to recipients least likely to survive.

The accuracy of prognostic one-year estimates at time of listing has also been called into question. This is particularly relevant not only for the recipient, but also for reportable quality metrics. Studies have shown that LAS often increases significantly between time of listing and time of transplantation, leading to falsely optimistic prognostication. Liu et al. identified that 12% of patients experienced a change of greater than 5 points in the LAS score, and this increased their risk of death by up to 30% [7]. Our own institutional experience has shown that up to 45% of recipients cross the threshold into an LAS of greater than 50 between the times of listing and transplantation. Thus, there is a need to provide recipients with updated prognostic summary.

Admittedly, the interplay of LAS and clinical acuity is a complex one. In our study, recipients with higher LAS groups differed from those with lower LAS by a trend towards higher requirement of ventilator and extracorporeal membrane oxygenation (ECMO) support. This is supported in previous analyses [6, 7, 9, 15]. The use of MV and ECMO is reflective of deteriorating clinical status and, by extension, worse survival. This was also true for extremes of BMI, GFR less than 60, diagnosis of pulmonary hypertension, and ICU admission. Increased LAS scores, however, drive impetus to proceed with LT despite the elevated risk of mortality because of the imminent or increasing risk of death. By disproportionately weighting LAS to focus on preventing waitlist death, the considerations of poor long-term survival appear to have been overlooked.

Shafii et al. challenged the utility of LAS following the observation that patients with higher scores had higher waitlist mortality and increased posttransplantation mortality [14]. In acquiescence, however, they concluded that, despite the higher mortality, it was not enough to warrant a departure from the current LAS system. Other reports have more pointedly criticized the current scoring system for the absence of weighting or use of indices which are otherwise strong predictors of mortality but yet paradoxically associated with lower LAS score. These include the use of ECMO and mechanical ventilation. Horai et al. postulated that the improved respiratory indices that accompany the use of mechanical support and ECMO lower the LAS scores and inaccurately skew the estimation of predicted survival in a more positive direction [8]. This is problematic because both ECMO and preoperative MV have been individually identified as powerful predictors of 1-year mortality [1, 16]. Horai et al. go even further to suggest that potential recipients with IPF should receive extra points, stemming from the observation of the rapid clinical deterioration of these candidates on the waiting list, with resulting increased risk of mortality.

Furthermore, the current scoring system does not take into consideration the contribution of donor lung quality. In face of organ scarcity, there is an increased impetus to use marginal donors. Recent analysis by Sommer et al. studied the influence of extended criteria donation on survival outcomes when marginal lungs were allocated to otherwise stable recipient [17]. Though this analysis was performed in Germany and prior to introducing LAS system, its results support the idea that organ allocation process should account for survival prognosis as a composite of both donor and recipient characteristics. Rather than merely ranking candidates on a waitlist, the scoring system should perhaps be matching the right recipients with the right donors.

There is thus a legitimate impetus to amend the current LAS system, to encourage the incorporation of ECMO and MV among others into the LAS, and to further reevaluate the contribution of primary pulmonary diagnoses and chronic comorbidities and donor characteristics so as to appropriately weight the score and more accurately predict survival risk. The fear that this might encourage use of a threshold beyond which LT would be precluded is theoretical and, as yet, unfounded [14]. In the context of allocation of scarce organs, however, it is likely to remain an ethical dilemma. As yet, however, no LAS score currently represents a prognosis so dismal as to pose a contraindication to LT. Nevertheless, Russo et al. recently calculated net survival as a composite outcome consisting of waitlist mortality and posttransplantation survival and concluded that the lowest net survival benefit is observed in groups with LAS <40 and >90, whereas the greatest survival benefit is observed with LAS ranging 50–80 [18].

Ideally, however, both recipients and payers should be provided with the most accurate estimates of survival commensurate with the clinical acuity best fitting the individual recipient. Perioperative expectations based on “best-case” survival estimates in pretransplant dialogue do not necessarily reflect those of recipients at the time of transplantation, often with recent escalations in LAS to scores above 50. The onus thus remains on the transplant center to convey acuity-specific prognostic information that best reflects the potential survival, based on the patients' most up to date clinical profile to allow for dynamic ongoing evaluation.

Our study has a number of limitations. As it pertains only to centers in the United States, it has limited generalizability to other centers in the world. It is likely that a handful of centers cater to the majority of recipients with high LAS scores and the results may reflect the experience of this smaller group of centers and not the entire cohort. The retrospective nature of the analysis and the reliance on registry data each increase the potential of both biases and threats from confounding factors. Though our study presents information previously proposed by others, its major contribution to the field lies in being the largest study to date to have evaluated the survival outcomes of patient based on LAS score alone, confirming the findings of several others and putting to rest the factual debate using national aggregate data. Furthermore, our risk factor analysis provides insights into where the LAS system may be most inadequate and provides theoretical suggestions as to how to improve the scoring system itself and to optimize its utility.

In summary, the LAS system disproportionately favors lowering waitlist mortality over long-term survival. Our current study highlights the impact of mortality risk factors that are not currently part of the risk calculus. Our results endorse the call for a shift to a scoring system that accounts for the presence of risk factors but also the dynamic clinical trajectory of potential recipients so as to better temper expectations and guide survival metrics based on LAS at the time of transplantation rather than that at listing.

## Figures and Tables

**Table 1:** Characteristics of patients at baseline, stratified by quartiles of LAS.

Recipient-related	Quartile 1	Quartile 2	Quartile 3	Quartile 4	*p* value
	(26.3–34.5)	(34.6–39.3)	(39.4–48.6)	(48.7–95.7)	
	*N* = 2576	*N* = 2576	*N* = 2576	*N* = 2576	
Age, years (SD)	57.3 (10.0)	53.3 (13.9)	53.8 (13.8)	54.6 (13.5)	<0.001
Male, *n* (%)	1,357 (52.7)	1,521 (59.1)	1,602 (62.2)	1,581 (61.4)	<0.001
White, *n* (%)	2,340 (90.8)	2,198 (85.3)	2,076 (80.6)	2,025 (78.6)	<0.001
Body mass index, Kg/m^2^ (SD)	25.2 (4.3)	24.5 (4.8)	25.2 (4.7)	25.2 (4.8)	
Diabetes, *n* (%)	240 (9.3)	450 (17.5)	527 (20.6)	608 (23.6)	<0.001
Diagnosis					
Idiopathic pulmonary fibrosis	153 (5.9)	741 (28.8)	1,297 (50.3)	1,531 (59.4)	<0.001
COPD/emphysema	1,800 (69.8)	760 (29.5)	185 (7.2)	100 (3.9)	
Cystic fibrosis	106 (3.3)	439 (17.0)	404 (15.7)	307 (11.9)	
Pulmonary hypertension	86 (16.7)	75 (2.9)	90 (3.5)	44 (1.7)	
Other	431 (16.7)	561 (21.8)	600 (23.3)	594 (23.1)	
Mechanical ventilator, *n* (%)	58 (2.3)	70 (2.7)	71 (2.8)	382 (14.8)	<0.001
ECMO support (%)	4 (0.2)	7 (0.3)	7 (0.3)	111 (4.3)	<0.001
GFR					
GFR <60	212 (8.2)	194 (7.5)	188 (7.3)	201 (7.8)	<0.001
60–90	965 (37.5)	911 (35.4)	894 (34.7)	747 (29.0)	
>90	1,399 (54.3)	1,471 (57.1)	1,494 (58.0)	1,628 (63.2)	
Chronic steroid use, *n* (%)	976 (37.9)	1,140 (44.3)	1,273 (49.4)	1,436 (55.8)	<0.001
Donor-related					
Age, years (SD)	34.6 (14.3)	33.9 (14.3)	33.8 (14.1)	34.4 (14.2)	0.13
Transplant related					
Admission to ICU, *n* (%)	52 (2.0)	68 (2.6)	86 (3.3)	566 (22.0)	<0.001
Graft ischemic time, hours (SD)	4.8 (1.7)	5.0 (1.6)	5.2 (1.7)	5.3 (1.7)	<0.001
Waiting time, days (IQR)	104 (33–310)	97 (29–269)	64 (22–205)	36 (10–130)	<0.001
Bilateral transplant (%)	1,581 (61.4)	1,679 (65.2)	1,662 (65.5)	1,833 (71.2)	<0.001

LAS: lung allocation score, *N*: number, SD: standard deviation, BMI: body mass index, COPD: chronic obstructive pulmonary disease, ECMO: extracorporeal membrane oxygenation, GFR: glomerular filtration rate, and ICU: intensive care unit.

**Table 2:** Comparison of survival rate after LT at 30 days, 90 days, 1 year, and 5 years.

	Quartile 1	Quartile 2	Quartile 3	Quartile 4
30 days	96.9%	96.8%	96.0%	94.8%
90 days	94.6%	93.7%	93.3%	90.9%
1 year	87.2%	85.0%	84.8%	80.9%
5 years	55.4%	54.5%	52.5%	48.8%

**Table 3:** Predictors of 5-year mortality after lung transplant.

Recipient demographic factors	Unadjusted		Adjusted	
	Hazard ratio (95% CI)	*p* value	Hazard ratio (95% CI)	*p* value
LAS				
Quartile 1 (26.3–34.5)	Reference (1)		Reference (1)	
Quartile 2 (34.6–39.3)	1.07 (0.97–1.18)	0.17	1.13 (1.01–1.26)	0.03
Quartile 3 (39.4–48.6)	1.11 (1.01–1.22)	0.04	1.17 (1.04–1.32)	0.01
Quartile 4 (48.7–95.7)	1.30 (1.18–1.44)	<0.001	1.17 (1.02–1.33)	0.02
Age, years	1.01 (1.00–1.01)	<0.001	1.01 (1.01–1.02)	<0.001
Male gender	1.11 (1.04–1.19)	0.004	1.10 (1.02–1.18)	0.01
Race, *n* (%)				
White	Reference (1)		Reference (1)	
Black	0.98 (0.86–1.11)	0.70	1.05 (0.92–1.19)	0.48
Hispanic	1.01 (0.87–1.18)	0.87	1.03 (0.88–1.21)	0.72
Asian	0.94 (0.73–1.21)	0.64	0.94 (0.72–1.21)	0.62
BMI				
Normal weight 18–24.9 kg/m^2^	Reference (1)		Reference (1)	
Underweight <18 kg/m^2^	1.18 (1.04–1.33)	0.01	1.30 (1.13–1.48)	<0.001
Overweight 25–30 kg/m^2^	1.11 (1.02–1.20)	0.011	1.02 (0.94–1.11)	0.64
Obesity >30 kg/m^2^	1.27 (1.15–1.41)	<0.001	1.16 (1.04–1.30)	0.001
*Recipient clinical acuity*				
Diabetes at transplant	1.02 (0.93–1.12)	0.66	0.98 (0.89–1.08)	0.68
GFR				
GFR <60	Reference (1)		Reference (1)	
60–90	0.75 (0.66–0.85)		0.77 (0.68–0.88)	<0.001
>90	0.68 (0.60–0.77)		0.72 (0.64–0.82)	<0.001
Type of transplant				
Unilateral	Reference		Reference (1)	
Bilateral	0.80 (0.74–0.86)	<0.001	0.79 (0.72–0.86)	<0.001
Primary pulmonary diagnosis				
Pulmonary fibrosis	Reference (1)		Reference (1)	
COPD	0.90 (0.82–0.98)	0.02	1.09 (0.97–1.22)	0.16
Cystic fibrosis	0.80 (0.71–0.90)	<0.001	1.16 (0.96–1.40)	0.11
Pulmonary hypertension	1.13 (0.92–1.37)	0.24	1.40 (1.14–1.73)	0.002
Other	0.88 (0.80–0.97)	0.01	1.06 (0.95–1.18)	0.95
ABO mismatch	1.16 (1.03–1.30)		1.17 (1.03–1.32)	0.01
Mechanical ventilation	1.58 (1.38–1.82)	<0.001	1.08 (0.92–1.28)	0.34
ECMO support	2.06 (1.53–2.77)	<0.001	1.34 (0.98–1.84)	0.06
Nonhospitalization	Reference		Reference (1)	
Non-ICU hospitalization	1.30 (1.15–1.47)	<0.001	1.36 (1.19–1.54)	<0.001
ICU hospitalization	1.97 (1.75–2.20)	<0.001	1.96 (1.69–2.27)	<0.001

Adjusted for graft ischemic time and waitlist duration in addition to variables in the table. LAS: lung allocation score, BMI: body mass index, COPD: chronic obstructive pulmonary disease, and ECMO: extracorporeal membrane oxygenation.
